# Iatrogenic Biliary Strictures: Surgical Experience with 39 Patients

**DOI:** 10.1155/1997/84803

**Published:** 1997

**Authors:** Luiz Rohde, Mário Sérgio Borges da Costa, Luis Roberto Wendt, Oly Corleta, Marcelo Ferreira

**Affiliations:** Department of Surgery Medical School of the Federal University of Rio Grande Do Sul Hospital De Clinicas Porto Alegre Brazil

## Abstract

The authors report their experience with surgical treatment of 39 patients with biliary strictures of iatrogenic origin. Patients were grouped according to the level of obstruction as described by Bismuth, and the type of repair was based on this classification. A total of 45 operations were performed, including those for recurrent strictures: 22 hepaticojejunostomies, 10 Hepp and Couinad's operations, 6 choledochojejunostomies, 3 separate right and left hepaticojejunostomies, 1 hepaticojejunostomy with mucosal graft (Smith's technique), 1 intrahepatic cholangiojejunostomy (Longmire's technique), 1 choledochoduodenostomy and 1 choledochoplasty. Results were considered good if the patient was free of symptoms, jaundice or episodes of cholangitis, with serum alkaline phosphatase less than two-times the normal value. Minimum follow-up period of two years (obtained in 35 patients) was required to evaluate the results. Good results were obtained in 26 of those 30 patients (87%) who underwent only one biliary reconstruction, and in 3 of those 5 (60%) with more than one repair. Overall, 29 patients (83% of those 35) presented good results. The complexity of the surgical treatment of biliary strictures imposes the adoption of measures to prevent lesions to the bile duct. Factors related to the prognosis that must be emphasized are surgeonsa' individual experience and skills, location of the stricture and diameter of the anastomosis.


*HPB Surgery*, 1997, Vol. 10, pp. 221–227
Reprints available directly from the publisher
Photocopying permitted by license only

© 1997 OPA (Overseas Publishers Association)
Amsterdam B.V. Published in The Netherlands
by Harwood Academic Publishers
Printed in India

### Iatrogenic Biliary Strictures: Surgical Experience with 39 Patients

LUIZ ROHDE, MÁRIO SÉRGIO BORGES DA COSTA, LUIS ROBERTO WENDT, OLY CORLETA and MARCELO FERREIRA

*Department of Surgery, Medical School of the Federal University of Rio Grande Do Sul, Hospital De Clínicas, Porto Alegre, Brazil*

*(Received 1 April 1995)*

**The authors report their experience with surgical treatment of 39 patients with biliary strictures of iatrogenic origin. Patients were grouped according to the level of obstruction as described by Bismuth, and the type of repair was based on this classification. A total of 45 operations were performed, including those for recurrent strictures: 22 hepaticojejunostomies, 10 Hepp and Couinad's operations, 6 choledochojejunostomies, 3 separate right and left hepaticojejunostomies, 1 hepaticojejunostomy with mucosal graft (Smith's technique), 1 intrahepatic cholangiojejunostomy (Longmire's technique), 1 choledochoduodenostomy and 1 choledochoplasty. Results were considered good if the patient was free of symptoms, jaundice or episodes of cholangitis, with serum alkaline phosphatase less than two-times the normal value. Minimum follow-up period of two years (obtained in 35 patients) was required to evaluate the results. Good results were obtained in 26 of those 30 patients (87%) who underwent only one biliary reconstruction, and in 3 of those 5 (60%) with more than one repair. Overall, 29 patients (83% of those 35) presented good results. The complexity of the surgical treatment of biliary strictures imposes the adoption of measures to prevent lesions to the bile duct. Factors related to the prognosis that must be emphasized are surgeons' individual experience and skills, location of the stricture and diameter of the anastomosis.**

*Keywords:* Biliary strictures, biliary obstruction, bile duct injuries, bilioenteric anastomosis

### INTRODUCTION

Recent studies show that accidental surgical lesions of the biliary ducts occur in 0.2% of open cholecystectomies [1,2] and that this percentage is higher with the laparoscopic technique [3,4]. These lesions can result in bile peritonitis, biliary fistula, biliary tract strictures or an association of these complications.

Repeated surgical procedures at the hepatic hilum makes the treatment of biliary strictures a great challenge, especially the higher ones, demanding skill and experience from the surgeon. Many surgical procedures have been described to treat these lesions [5–8], but the limited number of cases treated by the same surgeon makes the standardization of the reconstruction technique difficult. The classification of the biliary strictures proposed by Bismuth [9] has been adopted to describe these lesions. However, the important of its use in choosing the treatment has not been emphasized.

This study reports the experience of the Liver and Biliary Tract Surgical Group of the *Hospital de Clínicas de Porto Alegre* (HCPA) with the surgical treatment of iatrogenic biliary strictures in a 13

Correspondence to: Professor Luiz Rohde, Av. Palmeira 740, CEP 90470-300, Porto Alegre, Brazil.

year period, emphasizing the choice of technique according to the stricture level.

### PATIENTS AND METHODS

Thirty-nine patients with biliary strictures of iatrogenic origin were operated on by the authors between January 1980 and December 1993 at the HCPA. Twenty-three of these patients were female and 16 male. The average age was 45 years, with a range from 26 to 72 years. All but one patient were referred to HCPA from other hospitals, and no stricture followed laparoscopic cholecystectomy. The original procedures related to the biliary injuries are shown in Table I. In four patients, the injury was recognized during cholecystectomy and an attempt at repair effected. One patient with hepaticojejunostomy prior to referral had one retained stone. Jaundice was the main clinical feature, present in all patients, followed by abdominal pain in 36 (92%), fever in 29 (75%) and chills in 21 (54%). Mean serum alkaline phosphatase was 170 IU/L (range from 30 to 508 IU/L; normal values from 13 to 43), and the mean serum total bilirrubin was 146 µmol/1 (range from 34 to 578 µmol/l, normal values up to 17 µmol/l.

TABLE I Surgical procedures that led to biliary strictures

| Previous surgical procedure | *n* | % |
|---|---|---|
| Cholecystectomy | 27 | 69 |
| Cholecystectomy and exploration of the biliary ducts | 8 | 20 |
| Cholecystectomy and choledochoduodenostomy | 2 | 5 |
| Cholecystectomy and hepaticojejunostomy | 1 | 3 |
| Cholecystectomy and hepaticoduodenostomy | 1 | 3 |
| Total | 39 | 100 |

All patients underwent preoperative radiological studies to confirm the diagnosis and to define the location of the obstruction. Percutaneous transhepatic cholangiography (PTC) was performed in 33 patients and endoscopic retrograde cholangiopancreatography (ERCP) in 12 patients. Six of those 12 had both ERCP and PTC.

Patients were grouped according to the location of the stricture as described by Bismuth [9] (Fig. 1).

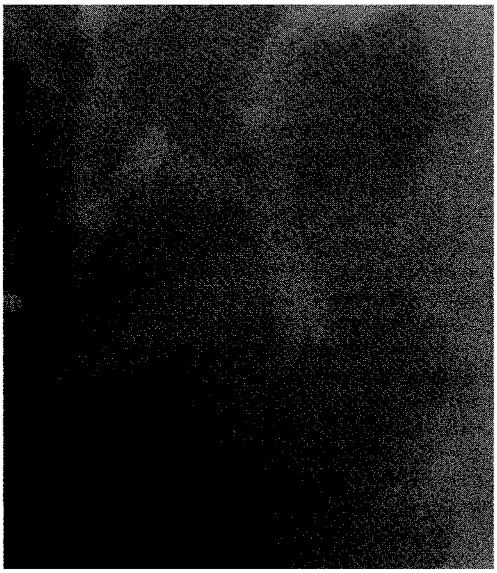

a

FIGURE 1 Distribution of the 39 patients according to the Bismuth classification. Type I: 8 cases.

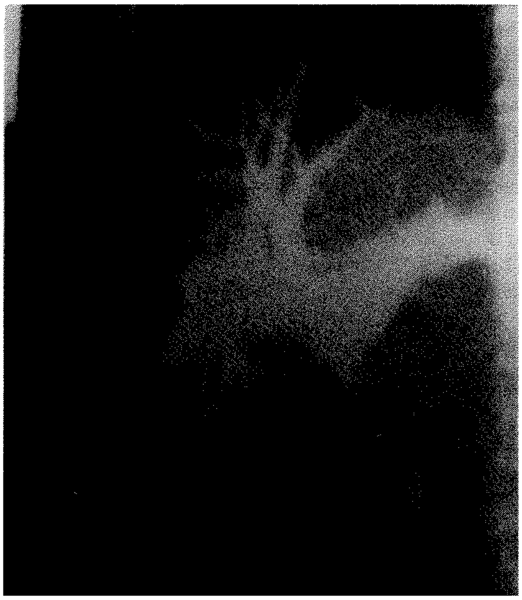

b

FIGURE 1 *Continued.* Type II: 22 cases.

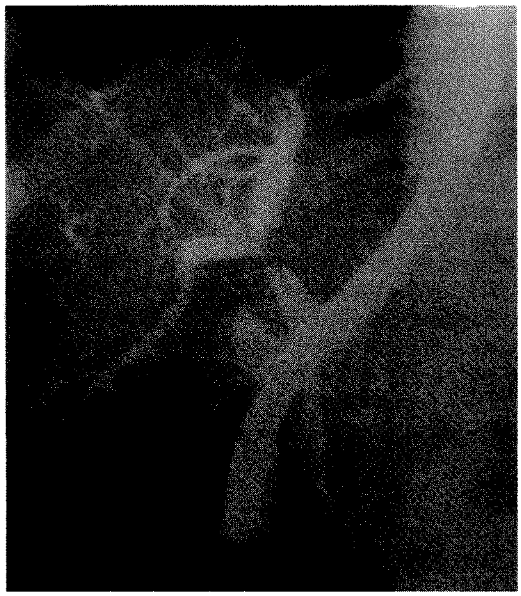

c

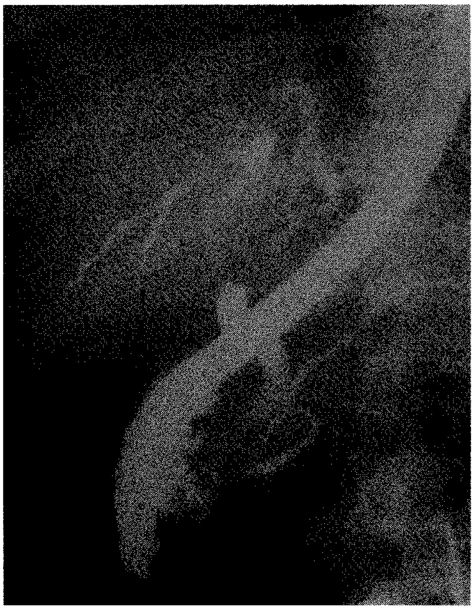

FIGURE 1 *Continued.* Type III: 7 cases d

FIGURE 1 *Continued.* Type IV: 2 cases

The initial surgical repair of these 39 patients is shown in Table II. All biliary-enteric anastomosis were performed with one-layer of interrupted stitches of chromic catgut 3–0 or

TABLE II Reconstructions used to treat the different types of strictures

| Type | Technique of reconstruction | *n* |
|---|---|---|
| I | Choledocojejunostomy* | 6 |
| | Choledochoduodenostomy | 1 |
| | Choledocoplasty | 1 |
| II | Hepaticojejunostomy* | 22 |
| III | Hepp & Couinaud* | 6 |
| | Hepaticojejunostomy* R and L | 1 |
| IV | Hepaticojejunostomy* R and L | 1 |
| | Hepaticojejunostomy* with mucosal graft (Smith) | 1 |
| Total | | 39 |

*Roux-en-Y reconstruction;R=right; L=left.

poligalactin (Vycril) 3–0, and those using jejunun were constructed in the Roux-en-Y fashion. Stents were used in 20 patients and left in place from 16 to 75 days (average of 56 days).

Results were considered good if the patient was free of symptoms, jaundice or episodes of cholangitis[10] with serum phosphatase less than twice the normal value [2,11,12]. Minimum follow-up period of two years was required to evaluate the results.

### RESULTS

A total of 45 biliary-enteric anastomosis were performed. Thirty-four patients underwent only one procedure. Five patients required more than one repair due to re-stenosis. In 4 of those 5 patients, stenotic hepaticojejunostomies were followed by Hepp and Couinaud's operations [6], one of which subsequently strictured and was repaired by Longmire's intrahepatic cholangiojejeunostomy [7]. The other patient with a stricture after Hepp and Couinaud's anastomosis required a separate right and left hepaticojejunostomy for repair. This patient has postoperative sepsis and was the only death of this series of 39 patients (2.5%). Nonlethal postoperative complications were wound infection (5 cases), external biliary fistula (4 cases with spontaneous closure), subhepatic abscess (1 case treated by percutaneous drainage) and intra-abdominal bleeding (1 case which demanded reoperation).

Reoperations after failure of the initial repair are shown in Table III. Thirty-five patients had a minimum follow-up period of 2 years, and the mean follow up period in this group was 37 months. Good results were obtained in 26 of those 30 patients (87%) who underwent only one biliary reconstruction, and in 3 of those 5 with more than one repair. Overall, 29 patients (83% of the 35) presented good results (Fig. 2). Previous repairs in those 4 patients with unsatisfactory results who underwent only one operation were one choledochoplasty, one choledochoduodenostomy, one separate right and left hepaticojejunostomy and one Smith's mucosal-graft [8]. The two patients with persistently elevated alkaline phosphatase did not undergo liver biopsy to rule out the development of cirrhosis.

TABLE III Reoperations after failure of the initial biliary repair based on Bismuth classification

| Type | *n* | Original surgery | Treatment (reoperation) |
|---|---|---|---|
| I | 0 | – | – |
| II | 4 | Hepaticojejunostomy | Hepp and Couinaud |
| III | 1 | Hepp and Couinaud | HJ* isolated R and L |
| | 1 | Hepp and Couinaud | Longmire |
| IV | 0 | – | – |

*HJ=hepaticojejunostomy; R=right; L=left.

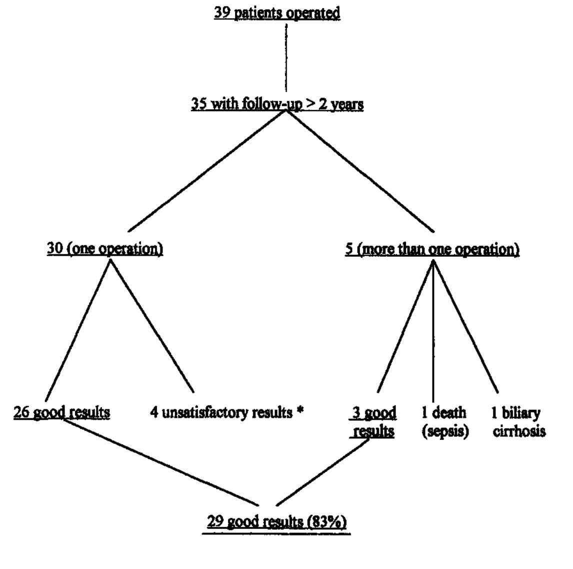

* 2 cases with episodic cholangitis and 2 cases with alkaline phosphatase persistently elevated.

FIGURE 2 Clinical result related to the number of surgical procedures.

### DISCUSSION

The great majority of the benign biliary strictures are secondary to accidental surgical trauma, and the operation that most often leads to these lesions is cholecystectomy [12, 13]. In this series, all referred patients have been submitted previously to open cholecystectomy, one third of them with associated bile duct exploration and four had undergone biliodigestive anastomosis. The author's experience in the treatment of biliary strictures induced by laparoscopic cholecystectomy began only after December 1993.

Videolaparoscopic cholecystectomy has become the treatment of choice of symptomatic cholelithiasis in many institutions, and a bile duct lesion is considered its most important complication [14]. The effect of this new approach in the incidence of biliary lesions is a cause of debate. In a North-American study of 1518 videolaparoscopic cholecystectomies [15], it has been observed that the frequency of intraoperative ductal lesions is greater in the first 13 case of the surgeon (2.2%), decreasing to levels comparable to open cholecystectomy with the increase of the surgeon's experience. In a study of 77,7604 cases treated in many institutions, Deziel and co-workers [16] observed that the mean rate of bile duct injury was 0.65% in hospitals performing 100 or fewer cases compared with 0.42% in centers that has performed more than 100 cases. However, these figures are well above those reported in open cholecystectomy. Recent publications demonstrate rates of bile duct injury from 0.16 to 0,2% [1,17]. Some authors suggest that laparoscopic cholecystectomy is associated with a inherently higher risk of bile duct injury than open cholecystectomy [3,4,16,18,19]. Besides, there was a seemingly increased number of patients with accidental biliary lesions referred to some institutions [4,14,18]. Measures to prevent biliary injury during laparoscopic cholecys-tectomy have been proposed by some authors [9–21].

Intraoperative recognition of the bile duct injury during the original operation occurred in only four patients of this series (10%). Most authors show intraoperative recognition rates during surgery of 12–22%, but in some series these rates reach up to 37– 42% [22, 23]. One possible explanation for the low frequency of early recognition of the biliary injuries in this and other series could be the fact that, in many cases, the bile duct wall is not sectioned, but the blood vessels are damaged. Northover and Terblanche [24] showed that the arterial supply of the supraduodenal duct is axial, with the main vessels being located at the 3 o'clock and 9 o' clock positions along the lateral and medial borders of the duct. These vessels are easily damaged, and there are reported cases confirming as ischemic basis for biliary strictures [25]. In order to avoid ischemic lesions, some authors advise minimum dissection of the proximal bile duct stump during reconstruction [5,26].

Thirty-three of 39 cases of the present series were investigated radiologically by percutaneous transhepatic cholangiography (PTC). This is considered the method of choice for radiological investigation in this setting because ensues visualization of the stricture location and the anatomy of the proximal ducts [10,12]. In addition, placing a catheter during the procedure allows preoperative external biliary drainage and facilitates intraoperative location of the bile duct proximal to the obstruction [10,13,27].

Transanastomotic tubes have been used by the vast majority of authors [5,8,10,13,22,23,28–31], although some use them selectively [9,11,12]. How long tubes should be kept in place is controversial. In general, it is recommended to maintain tubes for 3 to 6 months. In most complex cases, up to 12 months of stenting can be necessary [22,32,33]. The authors who use stents selectively consider that they are unnecessary if a tension-free anastomosis with wide stoma and good blood supply is accomplished. However, they use tubes in case of higher and more complex strictures, specially those with unsuccessful previous repairs. In this study, transanastomotic tubes were used selectively and were preferentially exteriorized through the excluded jejunal loop (like a Witzel jejunostomy) (Fig. 3), and exceptionally by the transhepatic route (Fig. 4). Data from this study (and other studies in which the selective use of tubes is adopted) don't permit valid conclusions concerning the influence of stents in outcome, since they were used only in complicated cases.

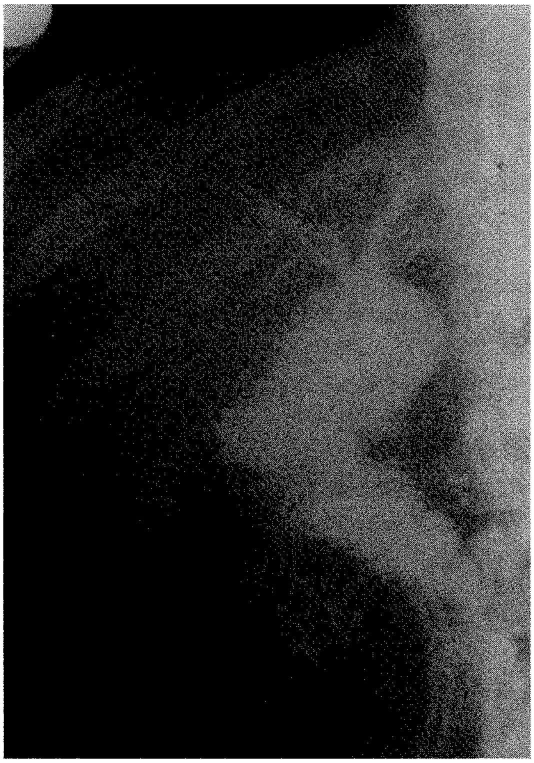

FIGURE 3 Cholangiography of a right and left hepaticojejunostomy with good result.

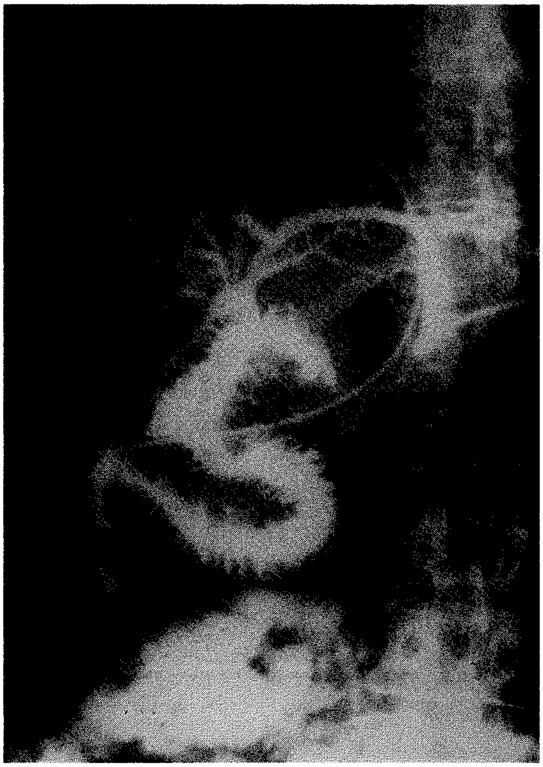

FIGURE 4 Cholangiography of a hepaticojejunostomy with mucosal graft (Smith's technique).

Caution is necessary to compare the results of surgical reconstruction from different institutions. Differences related to the location of the stricture, the moment of the repair (when the injury occurs or later on), the number of previous attempts of repairs, the presence of complications (biliary cirrhosis, portal hypertension, cholangitis), as well as diverse definitions of "good results" can lead to conflicting conclusions. One of the criteria to define "good results" in this study was alkaline phosphatase levels less than two times the normal values. Although it can be considered a rigid criteria, the authors think that it is valid since persistently elevated alkaline phosphatase levels denote some degree of cholestasis, and patients with this feature are at increased risk of developing cholangitis or biliary cirrhosis later on [11,12]. There was only one confirmed case of biliary cirrhosis (a patient with more than one repair). Two of the patients with one repair had persistently elevated alkaline phosphatase. Hepatobiliary scintigraphy (IDA scan) in one of these patients showed a delayed radionuclide passage to the jejunal loop indicating biliary stricture. None of these two patients underwent liver biopsies to investigate concomitant cirrhosis. The 83% of good results presented in this study is acceptable, since the literature shows figures from 70 to 90% [10].

The Bismuth classification has been used as a pattern to the description of biliary strictures. Analysis of the results of treatments based on this classfication has been used in the last years [2, 26, 30, 33] avoiding comparison between groups of patients with different levels of complexity. Based on the results of this and other sutides, the authors suggest the following standardization of the surgical treatment of iatrogenic bile strictures:

a) Type I: Roux-en-Y choledochojejunostomy or hepaticojejunostomy;
b) Type II: Roux-en-Y hepaticojejunostomy;
c) Type III: Roux-en-Y hepaticojejunostomy by Hepp and Couinaud's technique;
d) Type IV: repair according to the case (separate right and left hepaticojejunostomy or Smith's mucosal graft).

The length of choledochus and hepatic duct depends on the level of union of the cystic duct with the biliary tract: the higher the union between cystic duct and choledochus, the shorter hepatic duct is, making hepaticojejunostomy more difficult.

Choledochoduodenostomy was performed only once to hasten the operation of a high-risk elderly patient. Choledochoplasty was indicated only once too. Both techniques are not good for biliary stricture repair, and resulted in bad outcome in this series. The risk of poor results increases when the initial repair is inappropriate, the stricture is high and more than one reconstruction is necessary [27,33]. The occurence of two re-stenosis in 6 Hepp and Couinaud's operations does not mean that this technique is not valuable, but indeed denotes the complexity of the cases in which it was used (type III strictures).

The surgical treatment of iatrogenic biliary strictures is a great challenge. Even in experienced hands, significant morbidity and bad long-term results are frequent. Consequently, it is of utmost important to adopt routine measures to prevent accidental intraoperative bile dict injury [14,19–21]. During the operative repair, some principles of biliary reconstruction must be kept in mind, such as the importance of performing a wide tension-free anastomosis with mucosa-mucosa apposition in all circumference and with a good blood supply [26,27].

### *References*

[1] Roslyn, J.J., Binns, G.S., Hughes, E.F.X., Kirkwood, K.S., Zinner, M.J. and Cates, J.A. (1993). Open cholecystectomy: a contemporary analysis of 42, 474 patients. *Annals of Surgery*, **218**, 129–137.

[2] Raute, M., Podlech, P., Jaschke, W., Manegold, B.C., Trade, M. and Chir, B. (1993). Management of bile duct injuries and strictures following cholecystectomy. *World Journal of Surgery*, **17**, 553–562.

[3] Crist, D.W. and Gadacz, T.R. (1993). Complications of laparoscopic surgery. *Surgical Clinics of North America*, **73**, 265–289.

[4] Moosa, A.R., Easter, D.W., Van Sonnenberg, E., Casola, G and D'Agostino H. (1992). Laparoscopic injuries to the bile duct: a cause for concern. *Annals of Surgery*, **215**, 203–208.

[5] Bolton, J.S., Braasch, J.W. and Rossi, RL (1980). Management of benign biliary strictures. *Surgical Clinics of North America*, **60**, 313–332.

[6] Hepp, J and Couinaud, C. (1956). L'abord et l'utilization du canal hepatique gauche dans les reparations de la voie biliaire principale. *Presse Médical*, **64**, 947–948.

[7] Longmire, W.P. and Sandford, M.C. (1948). Intrahepatic cholangiojejunostomy withy partial hepatectomy for biliary obstruction. *Surgery*, **128**, 330–347.

[8] Wexler, M.J. and Smith, R. (1975). Jejunal mucosal graft: a sutureless technic for repair of high bile duct strictures. *American Journal of Surgery*, **129**, 204–211.

[9] Bismuth, H. (1983). Postoperative strictures of the biliary tract. In: *The Biliary Tract. Clinical Surgery International*, edited by L.H. Blumgart, **5**, 209–218. Edinburgh: Chruchill Livingstone.

[10] Lillemoe, K.D., Pitt, H.A. and Cameron, J.L. (1990). Postoperative bile duct strictures. *Surgical Clinics of North America*, **70**, 1355–1380.

[11] Pellegrini, C.A., Thomas, M.J. and Way, L.W. (1984). Recurrent biliary stricture: patterns of recurrence and outcome of surgical therapy. *American Journal of Surgery*, **147**, 175–179.

[12] Innes, J.T., Ferrara, J.J. and Carey, L.C. (1988). Biliary reconstruction without transtransanastomotic stent. *American Surgeon*, **54**, 27–30.

[13] Roslyn, J.J. and Tompkins, R.K. (1991). Reoperation for biliary strictures. Surgical *Clinics of North America*, **71**, 109–116.

[14] Davidoff, A.M., Pappas, T.N. and Murray, E.A., *et al.* (1992). Mechanisms of major biliary injury during laparoscopic cholecystectomy. *Annals of Surgery*, **215**, 196–202.

[15] The Southern Surgeons Club (1991). A prospective analysis of 1, 518 laparoscopic cholecystectomies performed by Southern U.S. surgeons. *New England Journal of Medicine*, **324**, 1073–1078.

[16] Deziel, D.J., Millikan, K.W., Economou, S.G., Doolas, A., Ko, S.T. and Airan, M.C. (1993). Complications of laparoscopic cholecystectomy: a national survey of 4,292 hospitals and analysis of 77,604 cases. *American Journal of Surgery*, **165**, 9–14.

[17] Morgenstern, L., Wong, L. and Berci, G. (1992). Twelve hundred open cholecystectomies before the laparoscopic era: a standard for comparison. *Archives of Surgery*, **127**, 400–403.

[18] Peters, J.H., Gibbons, G.D., Innes, J.T., *et al.* (1991). Complications of laparoscopic cholecystectomy. *Surgery*, **110**, 769–778.

[19] Rossi, R.L., Schirmer, W.J., Braasch, J.W., Sansers, L.B. and Munson, JL. (1992). Laparoscopic bile duct injuries: risk factors, recognition and repair. *Archives of Surgery*, **127**, 596–602.

[20] Hunter, J.G. (1991). Avoidance of bile duct injury during laparoscopic cholecystetomy. *American Journal of Surgery*, **162**, 71–76.

[21] Way, L. (1992). Bile duct injury during laparoscopic cholecystectomy (editorial). *Annals of Surgery*, **215**, 195.

[22] Browder, W., Dowling, J.B., Koontz, K.K. and Litwin, M.S. (1987). Early management of operative injuries of the extrahepatic biliary tract. *Annals of Surgery*, **205**, 649–658.

[23] Schulz, F., Függer, R., Herbst, F. and Huk, I. (1990). The therapy of iatrogenic lesions of the bile duct. *Hepato-Gastroenterology*, **37** (suppl II), 149–155.

[24] Northover, J.M.A. and Terblanche, J. (1979). A new look at the arterial supply of the bile duct in man and its surgical implications. *British Journal of Surgery*, **66**, 379–384.

[25] Terblanche, J., Allison, H. and Northover, J.M. (1983). An ischemic basis for biliary strictures. *Surgery*, **94**, 52–57.

[26] Csendes, A., Diaz, C., Burdiles, P., Nava, O., Yarmuch, J., Maluenda, F. and Fernandez E. (1992). Indications and results of hepaticojejunostomy in benign strictures of biliary tract. *Hepato-Gastroenterol*, **39**, 333–336.

[27] Moosa, A.R. (1990). Bile duct injury: some myths and realities. In *Progress in Hepatic, Biliary and Pancreatic Surgery*, edited by J.S. Najarian and J.P. Delaney, pp. 173–181. Chicago: Year Book Medical Publishers.

[28] Millis, J.M., Tompkins, R.K., Zinner, M.J., Longmire Jr, W.P. and Roslyn, J.J. (1992). Management of bile duct strictures: an evolving strategy. *Archives of Surgery*, **127**, 1077–1094.

[29] Muñoz, R., Cardenas, S. (1990). Thirty years' experience with biliary tract reconstruction by hepaticoenterostomy and transhepatic T tube. *American Journal okf Surgery*, **159**, 405–410.

[30] Pereira-Lima. (1992). Biliary reconstruction in benign postoperative stricture with transhepatic tubes. *American Journal of Surgery*, **164**, 124–128.

[31] Pitt, H.A., Miyamoto, T., and Parapatis, S.K., *et al.* (1982). Factors influencing outcome in patients with postoperative biliary strictures. *American Journal of Surgery*, **144**, 14–21.

[32] Pitt, H.A., Kaufman, S.L., Coleman, J., White, R.I. and Cameron JL. (1989). Benign postoperative biliary strictures: operate or dilate? *Annals of Surgery*, **210**, 417–427.

[33] Blumgart, L.H., Kellyer, C.J. and Benjamin, I.S. (1994). Bile duct stricture following cholecystectomy: critical factors in management. *British Journal of Surgery*, **71**, 836–843.

